# FixBox: A General Algorithm to Fix Molecular Systems in Periodic Boxes

**DOI:** 10.1021/acs.jcim.2c00823

**Published:** 2022-09-14

**Authors:** António M. Baptista, Lucie da Rocha, Sara R. R. Campos

**Affiliations:** Instituto de Tecnologia Química e Biológica António Xavier, Universidade Nova de Lisboa, Oeiras 2780-157, Portugal

## Abstract

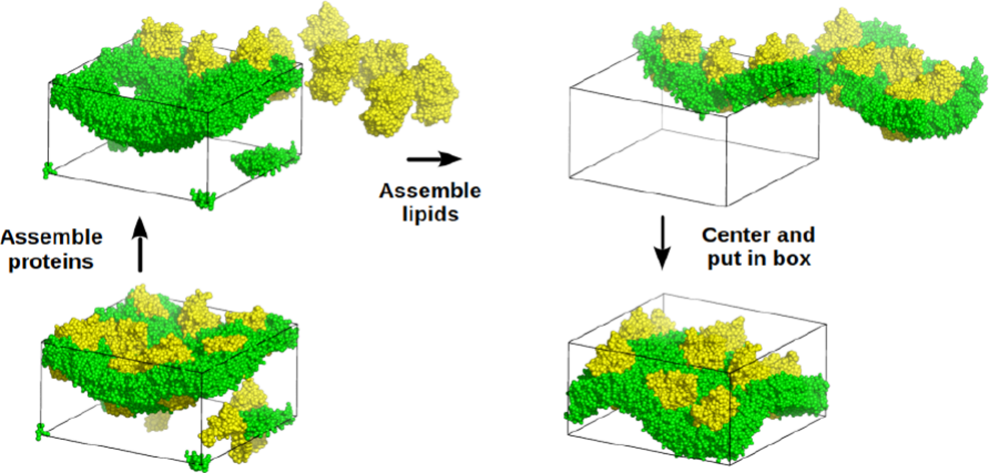

Periodic boundary conditions (PBCs) are a standard feature of molecular simulations, and their mathematical and computational aspects are well-understood and relatively straightforward. However, they can in practice be a nuisance when simulating heterogeneous systems, especially when different types of molecules change their relative positions during the simulation. Although the translation required to fix a broken molecular complex of interest can in most cases be easily inferred by visual inspection, it typically depends on the type of system, its configuration, and the box geometry, making automated procedures problematic. We present here a general algorithm, named FixBox, that can fix a molecular complex of interest from a minimal set of definitions of its assembling parts and intended arrangement in the simulation box. It uses a unified triclinic framework for the box geometric periodicity, does not require a full molecular topology, and is applicable to various types of systems and configurations, making it possible to fully and easily automate the fixing of a broken molecular complex. The performance of the algorithm is illustrated with problematic configurations of various types of simulated systems. The presented formal framework can generally be useful for algorithms that need to perform geometrical transformations on systems with PBCs.

## 1 Introduction

Molecular simulations are typically performed using periodic boundary conditions (PBCs), meaning that the simulation box can be regarded as surrounded by identical replica boxes in a face-to-face arrangement that fills the whole three-dimensional space.^1,2^ Thus, any atom that exits a box during a simulation is effectively entering into one of its contiguous boxes. Or, if we prefer to focus our attention on the *reference box* used by the simulation software being employed (typically having its center or one of its corners at the origin of the coordinates), any atom that exits the reference box through one of its faces can be regarded as effectively reentering through the opposite parallel face. Regardless of the interpretation, PBCs make possible the avoidance of surface effects caused by interaction with actual box walls and have become the standard approach for simulating condensed-phase systems.

However, although their mathematical and computational aspects are well-understood and relatively straightforward, PBCs can in practice be a nuisance. The problem is that the adoption of PBCs corresponds essentially to regard the system as homogeneous with respect to translations, a view that may not be the most suitable when dealing with heterogeneous systems in which some parts are inherently of more interest than others. In particular, in biomolecular simulations, there is typically some kind of molecular complex on which we want to focus our attention and which is initially centered or somehow properly positioned in the reference box; for example, a solvated multi-subunit protein would be well-assembled and placed at the box center, a membrane protein would be at the center of a horizontal membrane vertically aligned with the box midpoint, etc. However, as the simulation proceeds, the molecular complex may move across the box faces and end up no longer nicely encompassed within the reference box but rather “broken” across its faces; for example, a multi-subunit protein that moves to a box corner may end up with its subunits scattered at various box corners, a membrane bilayer that moves up or down may end up with its leaflets at opposite box sides, etc. Of course, this “breaking” with respect to the reference box is to be expected and can be dealt with transparently in some analyses by using *minimum image* (MI) distance vectors,^1,2^ but this is not always possible (e.g., the center of mass is ill-defined for a system with PBCs). In general, any procedure that requires the molecular complex to be a properly assembled object inside a single box, including simple visualization, becomes problematic.

**Figure 1:**
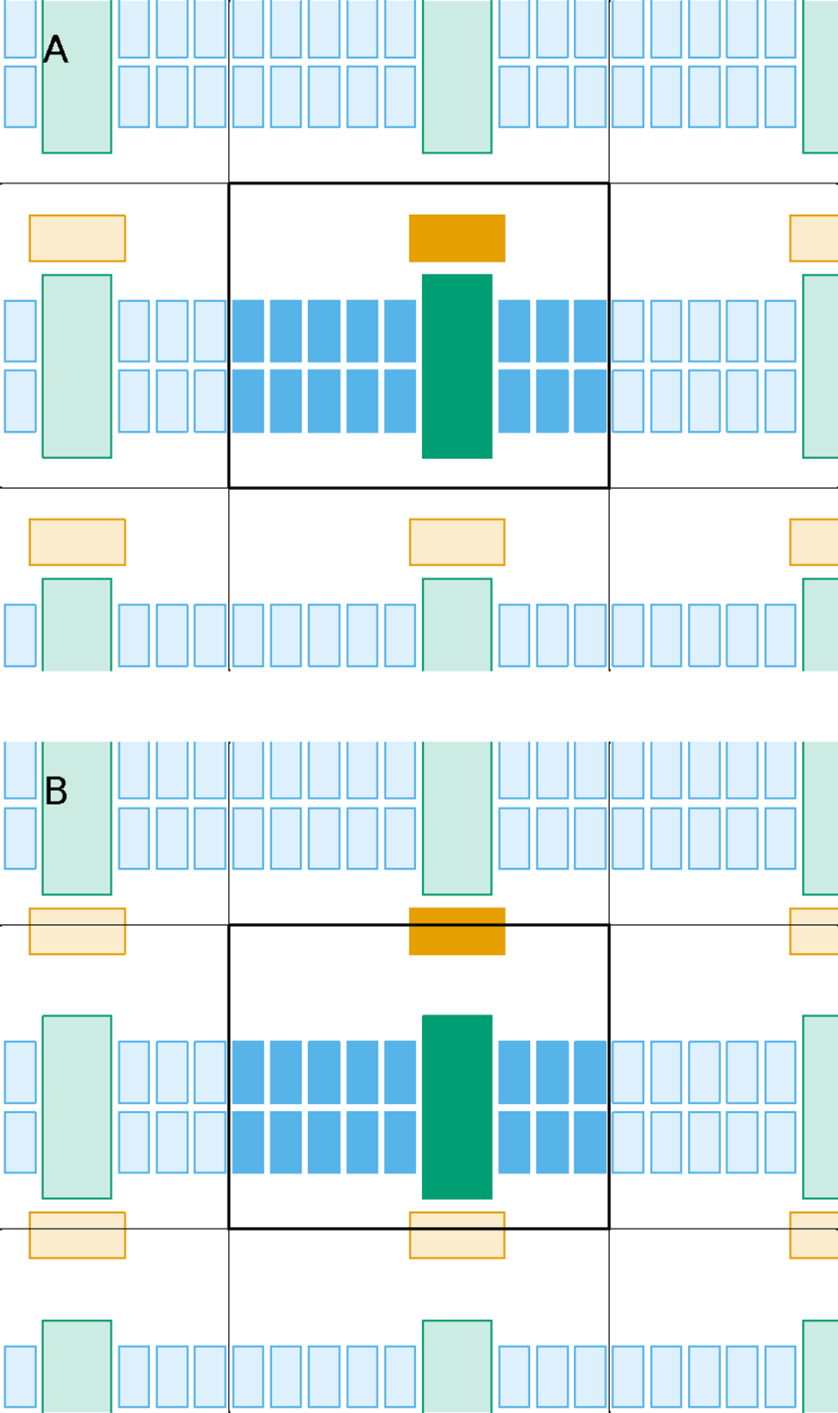
Scheme illustrating why fitting a system to a target configuration may fail when molecules change their relative positions. The scheme shows the reference box at the center and part of its surrounding replica boxes, depicting virtual replicas of the molecules as light-hued. Configuration A has the system well-assembled and centered in the reference box, being in principle a good target to which subsequent configurations can be fitted. If the orange molecule can transiently dissociate from the others during the simulation, we would typically restrict the fit to the blue and green molecules. However, even if those remain essentially unchanged and are nicely fit to configuration A, configuration B shows that the orange molecule may end up in the wrong position inside the reference box, i.e., dissociated instead of associated with the green molecule.

In order to fix the molecular complex, making it again nicely encompassed by the reference box, an obvious initial step is to keep molecular integrity. This can be easily done using the bond connectivity information and is often included in simulation software, which can generate coordinate files with whole molecules arranged in a simple way (e.g., with all molecular centers of mass inside the reference box). However, the resulting arrangement of the molecular complex, now consisting of whole molecules, will generally remain broken across the reference box. In order to finish fixing the system, we need to perform a translation that makes the complex to be nicely encompassed by the reference box while keeping PBCs. In fact, the required approximate displacement is usually quite obvious upon visual inspection (especially if one displays the box images), but determining the exact translation is in practice system-dependent and can seldom be obtained from a simple universal recipe. A simple standard approach is to start by superimposing the system on a previously built one in which the complex of interest was properly assembled and positioned in the reference box, but this can easily fail if some parts of the complex have changed their relative arrangement (Figure 1). Therefore, in practice one needs to devise a specific procedure for each system being studied, but even then it may fail for some configurations. This might seem a mere inconvenience when analyzing simulation data since it is usually easy to devise a specific procedure that would work for each occurring problematic configuration. However, this can become a rather serious problem when trying to implement automated methodologies (e.g., fixing a molecular complex before running Poisson–Boltzmann calculations, to be done periodically during constant-pH MD simulations^3,4^). Standard simulation packages often have tools that partially address this kind of problem (e.g., the option -pbc cluster in GROMACS’s trjconv program^5^ or the command autoimage in Amber’s cpptraj program^6^), but they usually require being used in combination with additional procedures (e.g., centering and application of PBCs) and depend on a full molecular topology that might be software-specific (meaning that using such a tool may require a full topology conversion); there are also some lipid-oriented tools that can identify distinct lipid aggregates, but they are not intended to rearrange the system into the reference box.^7,8^ Therefore, it would be convenient to have a more general and easy-to-use algorithm.

Although one generally wants the molecular complex of interest to be centered in the reference box, the crucial step of an efficient algorithm is the complex assembly rather than its centering. In fact, a multi-part object is necessarily ill-defined when using PBCs, requiring a proper consideration of the MI proximity between its parts (this is particularly obvious when the object itself is periodic, such as a lipid bilayer). It turns out that, upon visual inspection, we can easily determine how the parts can be assembled in a proper way because we actually know which relations between the parts are sensible and which ones are not. Therefore, we mentally perform a sequence of sensible assembly operations (e.g., start by assembling the subunits of a protein, then assemble the whole protein with a nearby ligand, then assemble the lipids of the surrounding bilayer, etc.), and only after that do we think about the global translation needed to center the assembled complex into the reference box and, finally, about the individual PBC-complying translations that would bring each of the remaining molecules into the reference box. The algorithm proposed here, named FixBox, tries to simply follow the same sequence of geometric operations that we seem to instinctively perform in our mind when visually inspecting a simulation box: (1) evaluate the MI-proximities between the molecules constituting the molecular complex of interest; (2) assemble together those molecules based on the MI-proximities; (3) center the resulting assembled system in the reference box; and (4) apply any required individual translations to bring other molecules into the reference box. For simplicity, it is assumed that the provided atomic coordinates already preserve molecular integrity (which, as noted above, can be trivially generated by simulation software). In addition to proposing the FixBox algorithm, the present article provides a general formal/mathematical framework that can be useful for algorithms that perform geometrical transformations on systems with PBCs; so far, the description of such algorithms has mostly been limited to textual descriptions or to the actual source code, which can lead to misunderstandings or be impractical.

## 2 Theory and Methods

### 2.1 Triclinic Boxes

There are only five possible types of space-filling convex polyhedra that can be used as simulation boxes with PBCs, the so-called parallelohedra: parallelepiped, rhombic dodecahedron, truncated octahedron, hexagonal prism, and rhombo-hexagonal dodecahedron.^9^ Of these, only some regular forms of the parallelepiped (cube and right-rectangular prism), the regular rhombic dodecahedron, and the regular truncated octahedron are common as simulation boxes.^2,10^ However, the resulting five types of space-filling arrangement of polyhedra are just alternative ways of splitting the space, and they can all be expressed in terms of the more general parallelepipedic or *triclinic* lattice.^11^ Therefore, for simplicity and uniformity of treatment, it is assumed here that the reference box is parallelepipedic/triclinic; transformation to and from the more traditional box shapes can be done using available software tools (e.g., the GROMAC’s trjconv program^5^).

A triclinic lattice is fully defined by a set of three linearly independent vectors **b**_1_, **b**_2_, and **b**_3_, called *primitive vectors*.^12^ (The term “triclinic” is often used in crystallography only for lattices for which these vectors have different lengths and different angles between themselves, but no such restriction is adopted here.) Given an initial point, the primitive vectors can be used as directed edges that generate the triclinic reference box (yielding its other seven corners by adding, respectively, **b**_1_, **b**_2_, **b**_3_, **b**_1_ + **b**_2_, **b**_1_ + **b**_3_, **b**_2_ + **b**_3_, and **b**_1_ + **b**_2_ + **b**_3_ to that initial box corner), whose replication generates the whole infinite lattice of stacked boxes (by applying the translation vector *k*_1_**b**_1_ + *k*_2_**b**_2_ + *k*_3_**b**_3_ for all ). The relation of this lattice scaffold to the Cartesian reference frame depends on the particular implementation, the two most common choices being perhaps to define the reference box to have its geometric center at either **g** = (**b**_1_ + **b**_2_ + **b**_3_)/2 or **g** = 0. It is convenient to choose the primitive vectors with the same handedness as the Cartesian reference frame, in which case **b**_1_·(**b**_2_ × **b**_3_) is positive for a right-handed frame and negative for a left-handed one; the absolute value of this triple product is the volume of the triclinic box.^1,13^

It is important to keep in mind that the triclinic reference box thus defined is simply a convenient way to represent the periodic system embedded in the lattice. For a given lattice, there are infinitely many ways of splitting the whole space into periodically arranged identical *primitive unit cells*, whose shape is largely arbitrary (including non-convex shapes), and even a triclinic shape is non-unique due to the infinitely many ways of selecting a valid set of primitive vectors.^11,12^ Therefore, primitive vectors should be chosen in a sensible way, avoiding unnecessarily skewed or elongated reference boxes that may fail to encompass the molecular complex of interest; sensible choices are available for the usual regular parallelohedra (e.g., see Table 5.6 in ref (5)) and can also be devised for irregularly shaped boxes (in which case lattice reduction^14^ might be useful). In some cases, though, it may be impossible to keep the whole complex inside a reference box that is otherwise convenient for the system being analyzed (see step 4 of the algorithm in section 2.3). In any case, it is assumed here that a convenient set of primitive vectors is provided, regardless of whether the corresponding triclinic reference box initially encompasses the system molecules or not (e.g., they may be initially arranged in a rhombic dodecahedral box).

### 2.2 Scaled Coordinates

A point in physical three-dimensional space is usually written as a position vector and expressed in terms of Cartesian coordinates, **r** = (*r*_*x*_, *r*_*y*_, *r*_*z*_). When dealing with PBCs, it is convenient to adopt a set of *scaled coordinates* (also called *reduced coordinates*) that can simplify many calculations and geometric transformations. A position vector **r** is related to the vector **s** of its scaled coordinates as^1,13^
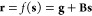
1where **g** is the geometric center of the reference box and **B** is the *box matrix* obtained by collecting the primitive vectors as column matrices
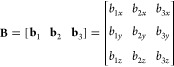
2The determinant of **B** is equal to **b**_1_·(**b**_2_ × **b**_3_), whose absolute value is, as noted above, the box volume. Therefore, **B** has a non-null determinant and is thus invertible, giving
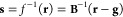
3If, instead of a position vector, we consider a free vector **r**_*AB*_ pointing from point *A* to point *B*, we get
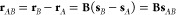
4and **s**_*AB*_ = **B**^–1^**r**_*AB*_, where we defined **s**_*AB*_ = **s**_*B*_ – **s**_*A*_; thus, **g** is dropped when mapping free vectors.

The treatment of PBCs becomes straightforward in terms of scaled coordinates, being convenient to define the nearest integer from a real number *a*, ⌈*a*⌋ = ⌊*a* + 1/2⌋, where ⌊·⌋ denotes the floor function,^15^ and its vectorial counterpart  for a vector **a** = (*a*_*x*_, *a*_*y*_, *a*_*z*_). If we consider an atom *i* at **r**_*i*_, with scaled position **s**_*i*_ = *f*^–1^(**r**_*i*_), it is easy to see that atom *i* is inside a box that is ⌈*s*_*i*,α_⌋ boxes away from the reference box in each of the directions α = *x*, *y*, *z* and that the “local” position of that point in its box is identical to that of the point
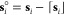
5in the reference box, whose coordinates are always in the interval [−1/2, 1/2); for example, **s**_*i*_ = (2.5, 5.2, −3.6) gives ⌈**s**_*i*_⌋ = (3, 5, −4) and **s**_*i*_^°^ = (−0.5, 0.2, 0.4). Similarly, if **s**_*ij*_ = **s**_*j*_ – **s**_*i*_ is the vector between atoms *i* and *j*, the MI vector between them is
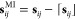
6(unless *i* and *j* are too far apart; see below). The counterparts in the physical triclinic lattice can then be obtained by using eq 1 for position vectors and (4) for free vectors
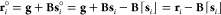
7and
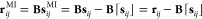
8These relations are included here for convenience, being well-known in molecular simulation.^1,13^ The relations (6) and (8) for the MI distances are valid only if the reference box is rectangular or the length of the **r**_*ij*_^MI^ thus computed does not exceed half of the shortest primitive vector (e.g., see the discussion and Figure 3.13 on page 71 of ref (16)), which means that these relations are usually safe to use when searching for pairs of nearest molecules (see section 2.3).

The change from physical Cartesian coordinates to scaled ones corresponds to a geometric transformation that maps the physical triclinic lattice to a non-physical lattice of unit cubes (i.e., with side equal to 1 unit), with the original reference box being mapped to the cube centered at the origin. Since **B** contains the actual length values of the primitive vectors (e.g., in nanometers), this cubic lattice is dimensionless, with **s** = (*s*_*x*_, *s*_*y*_, *s*_*z*_) being a triplet of bare real numbers, where the subscripts *x*, *y*, and *z* refer to the cubic lattice axes and not to the original ones in physical space. The mappings *f* and *f*^–1^ are affine transformations, which preserve the following geometric features:^17^ linearity and planarity, parallelism and intersection of lines and planes, and ratios of lengths between parallel line segments. As shown below, many transformations involving a triclinic lattice can be entirely conducted in the scaled cubic lattice, with the back-mapping taking place only at the end.

### 2.3 FixBox Algorithm

#### Step 1: Measure Intermolecular Proximity

We want a measure of molecular proximity that retains some convenient properties of the physical-to-scaled transformation of coordinates, particularly eq 4. If we designate the distance vector between two molecules *M* and *N* as **R**_*MN*_ in physical space and as **S**_*MN*_ in scaled space, we want to have **R**_*MN*_ = **BS**_*MN*_ so that we may use an equation analogous to (8), namely,
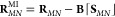
9and thus simplify the treatment of PBCs for molecules. Although this equation inherits the limitation already noted apropos of eq 8, intermolecular distances will be used only for selecting pairs of nearest molecules (see step 4), making that limitation irrelevant in practice.

**Figure 2:**
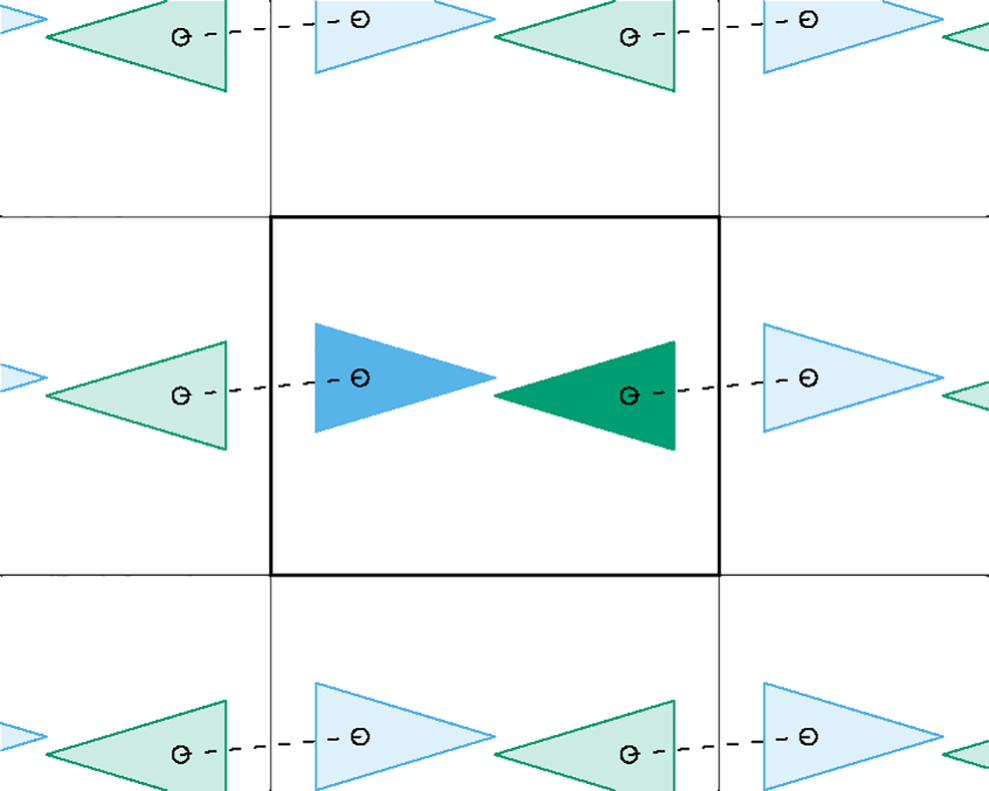
Scheme illustrating why the distance between molecular centers can be a bad measure of intermolecular distance. Even though the tips of both molecules are very close to each other at the center of the box, the use of the molecular centers (small circles) would lead us to take the dashed segment as the shortest distance between them.

One candidate for **R**_*MN*_ is the distance vector between the two molecular centers. The center of a molecule *M* can be generally defined as a weighted sum over the positions of all atoms *i* ∈ *M*
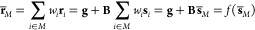
10with ∑_*i*∈*M*_*w*_*i*_ = 1, which shows that the physical and scaled centers are directly mapped to each other. The exact nature of the center  depends on the weights *w*_*i*_: it is the geometric center of *M* if *w*_*i*_ is the same for all atoms *i* ∈ *M*; it is the center of mass of *M* if *w*_*i*_ is the fraction of molecular mass contributed by *i* ∈ *M*; etc. The distance vector between the centers of *M* and *N* is then
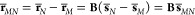
11which suggests that  and  might be good definitions for **R**_*MN*_ and **S**_*MN*_. However, this can easily lead to problems with asymmetric molecules: for example, if two closely interacting monomers have their bulkier parts away from their physical interface,  may point away from that interface, connecting two monomers that would be visually interpreted as obviously belonging to different lattice images of the dimer (Figure 2).

Another candidate for **R**_*MN*_ is the interatomic distance vector that yields the shortest MI-distance between the two molecules. Thus, we can define an intermolecular distance vector
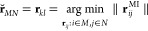
12and its scaled counterpart , which obviously satisfy the intended relation . (Note that, in contrast to **r**_*kl*_ in physical space, in general, **s**_*kl*_ does *not* have the shortest MI-length among the interatomic vectors in scaled space because affine transformations preserve ratios of lengths only between parallel line segments.) The calculation of the  pairs scales with the square of the total number of atoms, while that of the  pairs scales linearly with this number. However, in practice,  seems to always agree with our intuitive sense of molecular proximity and avoids the above-mentioned problems associated with using . Therefore, although computationally less efficient,  seems a better choice for **R**_*MN*_ than . In what follows, we keep the treatment general, simply requiring that **R**_*MN*_ = **BS**_*MN*_. We note also that ∥**R**_*MN*_∥ is a measure of molecular proximity but *not* necessarily a metric (in the sense used in topology^18^); for example, the triangle inequality ∥**r̆**_*MN*_∥ ≤ ∥**r̆**_*MP*_∥ + ∥**r̆**_*PN*_∥ may not hold for some molecules *M*, *N*, and *P*.

#### Step 2: Assemble the Molecular Complex

There is usually a set of molecules that we identify as composing the molecular complex that we want to assemble. The second step of the algorithm consists in moving those molecules to new positions that, together, preserve the wholeness of that complex. This will generally imply translating each molecule by a different displacement that corresponds to an integer number of boxes in each lattice direction. If we denote as  the set of molecules to be assembled, our aim is then to find an integer-valued displacement vector **K**_*M*_ for each molecule , such that the new atomic positions in scaled space
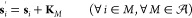
13will preserve the complex wholeness. This gives
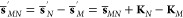
14where we used the definition of  in eq 10 and
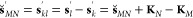
15where *k* ∈ *M* and *l* ∈ *N* are the minimum-distance atoms defined in eq 12. Therefore, we have
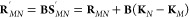
16whether we define **S**_*MN*_ as  or as  (see step 1 of the algorithm).

Now, given any two molecules *M* and *N* in , it may seem reasonable to require that, after the translations, they are as close as possible to each other, that is, that **R**_*MN*_^′^ = **R**_*MN*_^MI^. Using eqs 9 and 16, this would imply the integer relation
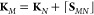
17However, if this equation were to hold for any pair of molecules, any three molecules would have to satisfy ⌈**S**_*MN*_⌋ = ⌈**S**_*MP*_⌋ + ⌈**S**_*PN*_⌋, which is generally impossible: for example, **S**_*M*_ = (−0.3, 0, 0), **S**_*P*_ = (0, 0, 0), and **S**_*N*_ = (0.3, 0, 0) give ⌈**S**_*MN*_⌋ = (1, 0, 0) but ⌈**S**_*MP*_⌋ + ⌈**S**_*PN*_⌋ = (0, 0, 0). Therefore, we require instead that eq 17 holds only for neighboring molecules, starting with a nucleation point and then gradually moving molecules to their new positions, computing the displacement vector **K**_*M*_ of each added molecule *M* from the **K**_*N*_ of a previously assembled neighboring molecule *N*. More exactly, the assembled complex is built from its initially non-assembled molecules using the following sequential procedure:

(a) Select an initial molecule , set **K**_*P*_ = **0** and mark *P* as assembled
(b) Select the pair of molecules with the lowest ∥**R**_*MN*_^MI^∥, where *M* is unmarked and *N* is marked as assembled and then compute **K**_*M*_ with eq 17 and mark *M* as assembled;
(c) Repeat step (b) until all molecules in  are marked as assembled.
(d) Apply eq 13 to all atoms of molecules in , otherwise (e.g., for the solvent) keep their original positions, that is, **s**_*i*_^′^ = **s**_*i*_.

**Figure 3:**
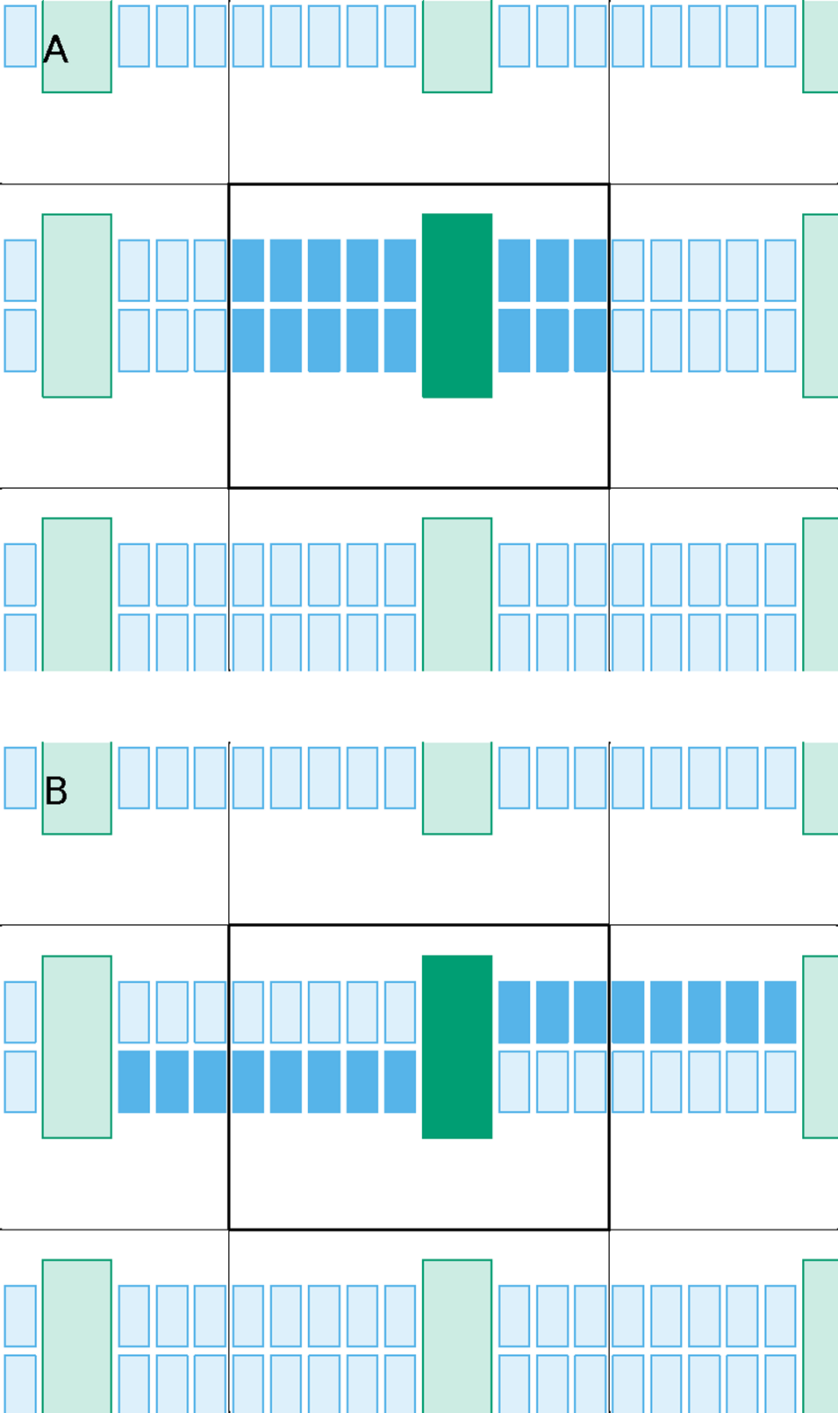
Scheme illustrating that the assembly algorithm can spread the system over several boxes. If the blue molecules represent lipids in a bilayer, it turns out that their nearest neighbor usually resides in the same monolayer (as illustrated in section 3). Therefore, even when the system is already well assembled in the reference box (A), the assembly algorithm may end up placing the monolayers away from each other in the resulting nearest-neighbors tree (B).

This will produce a complex having its molecules assembled by MI pairwise proximity, thus producing a tree (in the graph-theoretic sense^19^) that is independent of the initially chosen molecule. However, this assembled tree will be in general not centered nor fully contained in the reference box, possibly overlapping several of its neighboring boxes. In particular, if the molecular complex includes intrinsically periodic parts composed of small molecules (e.g., a lipid bilayer), its molecules tend to get scattered over a substantially large region, possibly larger than a single box (Figure 3). This is not a problem, though, being addressed in step 4. We note also that, since intermolecular distances are used only to select pairs of nearest molecules, in step (b), the already mentioned limitation of eq 9 becomes in practice irrelevant, especially when using .

**Figure 4:**
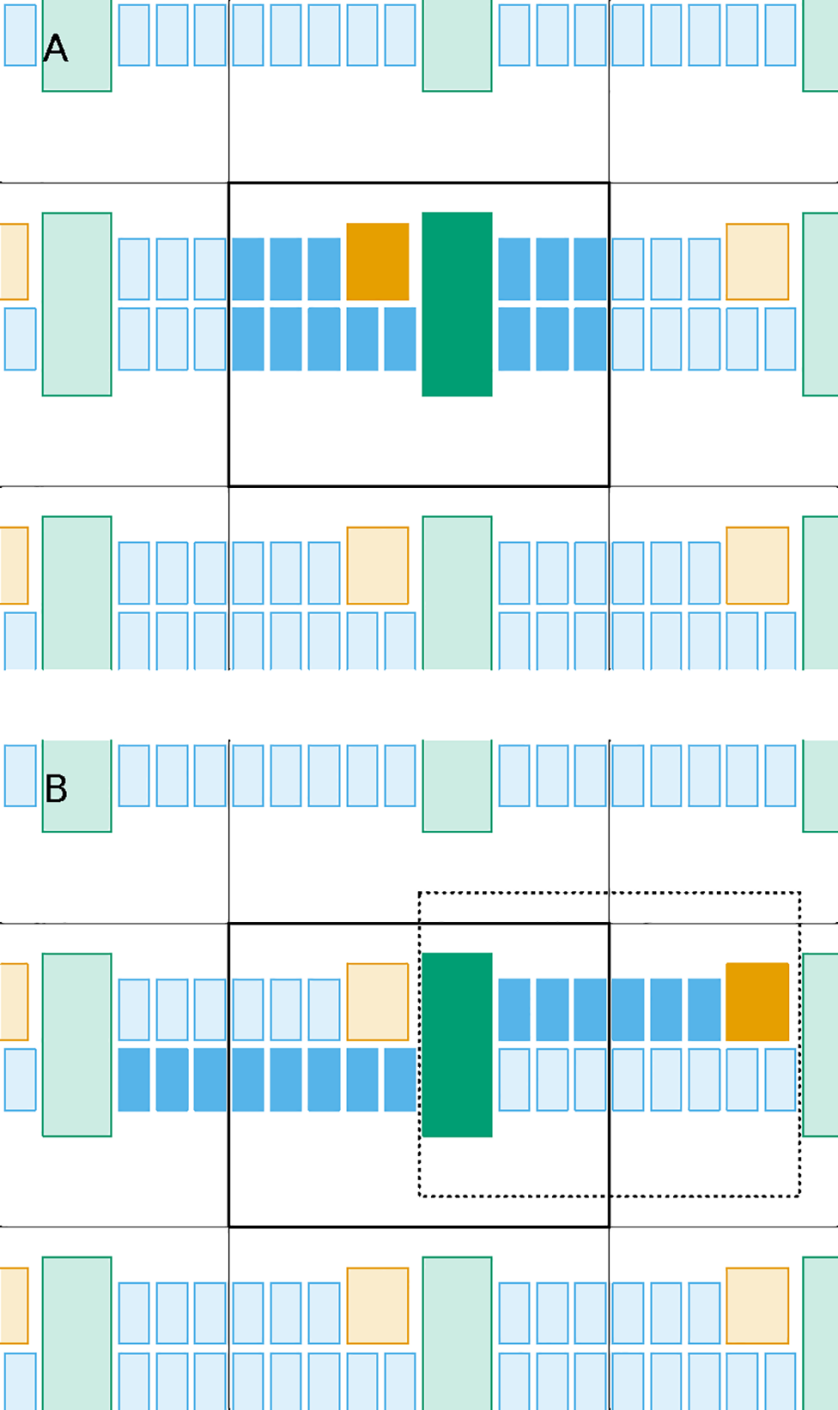
Scheme illustrating that a single-stage assembly can set apart molecules that we want to keep together. If the nearest-neighbor of the orange molecule is a blue molecule, the effect already described in Figure 3 can be observed; thus, even when the system is already well assembled in the reference box (A), a single-stage assembly may end up placing the orange and green molecules away from each other (B). This would effectively break the green–orange complex, which would be interpreted as encompassed by the dashed box shown in (B).

In some cases, it may be convenient to split the assembly process in several successive stages. For example, if the complex consists of a lipid bilayer with an embedded multi-subunit protein, we naturally want to keep the protein whole, but the algorithm just described may end up connecting different images of some subunits through a chain of lipid molecules that happen to be all particularly close to each other, thus placing some subunits separated from each other (Figure 4); in this case, it would be convenient to first assemble the protein subunits and then proceed by assembling the lipid molecules on top of the already assembled protein. Therefore, instead of following the single sequence (a–d) indicated above, it is often convenient to split  into a collection of disjoint subsets of molecules, , and proceed in stages: first follow (a–c) to assemble the molecules in , then repeat (b,c) to assemble the molecules in  on top of the already assembled set , then repeat (b,c) to assemble the molecules in  on top of the already assembled set , etc., until all molecules in  are assembled, and then apply (d) to compute the new positions. This multi-stage approach provides further control of the assembly process, making it possible to enforce the chemically sensible molecular complex.

#### Step 3: Center the Molecular Complex

**Figure 5:**
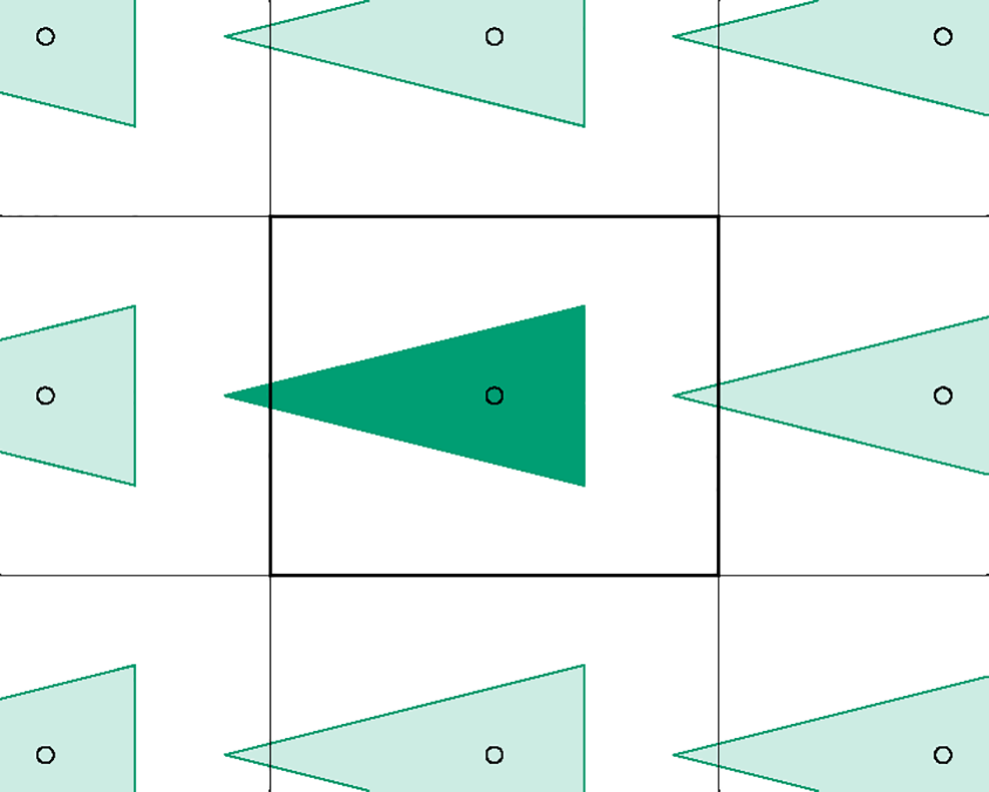
Scheme illustrating that moving the center of a molecule (small circle) to the center of the reference box may result in poor centering for an asymmetric molecule.

**Figure 6:**
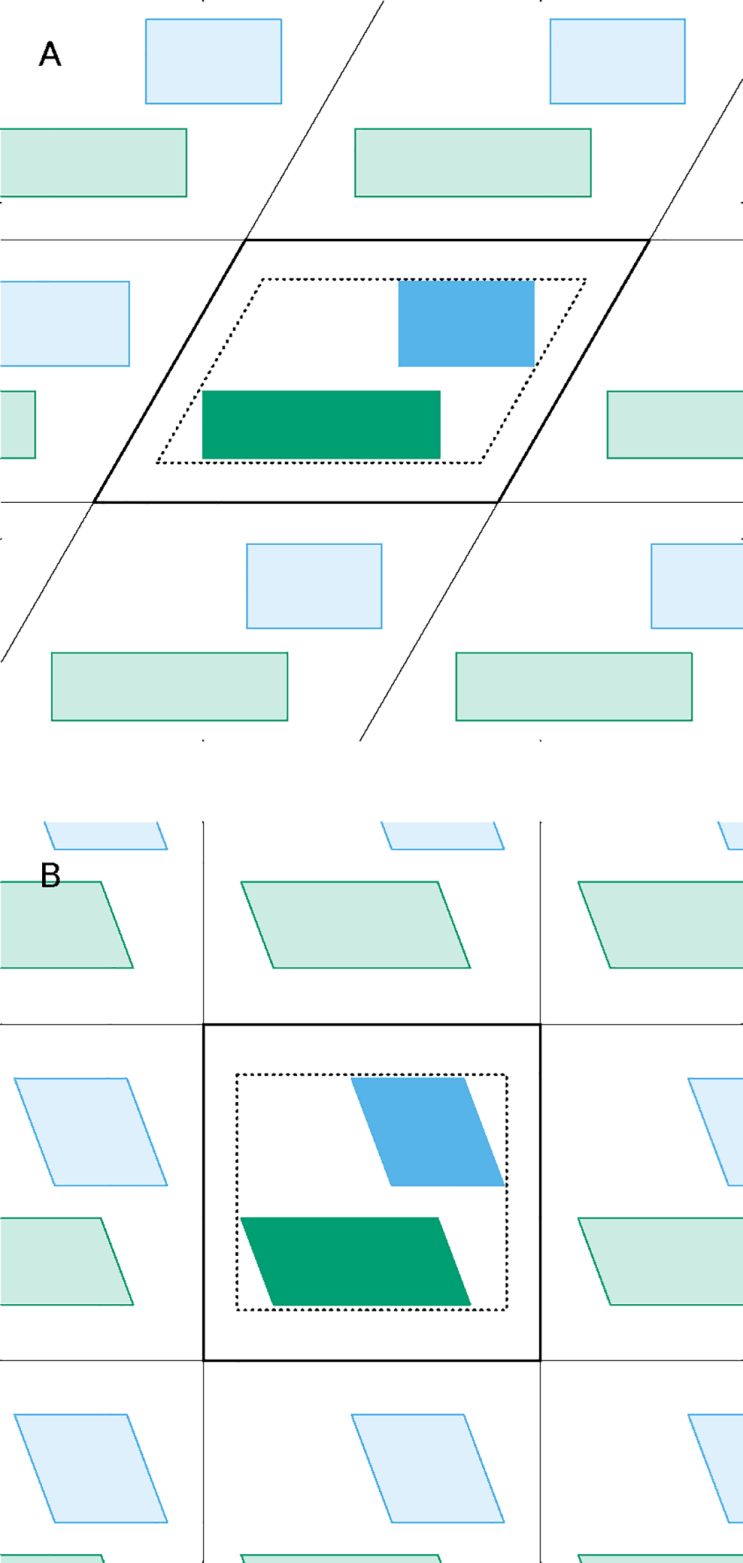
Scheme illustrating that centering the bounding box (dotted line) of a set of molecules in physical space (A) is equivalent to center their bounding box in scaled space (B).

The next step is to center the system assembled in step 2 in the reference box. Since, as seen above, the assembly algorithm may leave some parts of the system still scattered across multiple boxes, it is convenient to center not  itself but rather a subset  of the molecules of  that we know to be already in their final relative arrangement (ensured through the definition of ). One possibility is to simply translate the molecular center of  (eq 10) to the center of the box, but this may fail for a highly asymmetric complex, leaving part of its periphery sticking out of the box (Figure 5). A better alternative is to center the *bounding box* of , defined as the smallest triclinic box that fully encompasses  and has its four faces parallel to those of the reference box; thus, the whole assembled system must experience a translation that moves the geometric center of this bounding box to that of the reference box or, equivalently, that places opposite faces of the bounding box equidistant to the corresponding reference box faces. This is most easily done in scaled space, taking advantage of the properties of affine transformations. Since parallel planes are preserved, the faces of the bounding box in scaled space will remain parallel to the reference box faces when transformed to physical space. Furthermore, the fact that length ratios on a line are also preserved implies that, if an edge of the bounding box is equidistant to a pair of parallel faces of the reference box in one space, it will remain equidistant in the other space. Therefore, a centered bounding box in scaled space is transformed to a centered bounding box in physical space (Figure 6), meaning that the required translation can be done entirely in scaled space. The centered bounding box thus obtained might in some cases protrude out of the reference box along one or more directions, which can sometimes be successfully addressed, as discussed in step 4.

For greater flexibility, it is convenient that different parts of the system may be used for centering along different directions: for example, for a protein embedded in a horizontally oriented lipid bilayer, we typically want to use only the protein for the horizontal centering (thus placing the protein at the center of the bilayer horizontal projection) and use the whole complex for the vertical centering. Thus, instead of using a single set , we generally want to choose three sets of molecules for centering in each direction, , , and , which would be typically subsets of . The procedure will then be to define the bounding box by finding the atoms with the minimum and the maximum coordinate values along each direction, using the **s**_*i*_^′^ positions from step 2, and then translate its center to the center of the reference box (which in scaled space is at the origin). Therefore, we define the vectors **s**_min_^′^ and **s**_max_^′^ with components
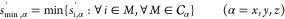
18and
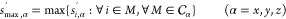
19and then apply to all atoms of the system a translation that moves the center of the bounding box to the origin in scaled space, thus producing the new atomic coordinates
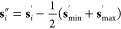
20The system thus obtained should have the subsets , , and  assembled and centered in the reference box. As after step 2, if the complex includes intrinsically periodic parts composed of small molecules (e.g., bilayer lipids that were most certainly not part of the  subsets), some of these molecules may still remain outside of the reference box, at least partially; this is fixed in step 4.

#### Step 4: Place the System inside the Reference box

As noted above, when the molecular complex includes intrinsically periodic parts composed of small molecules (e.g., a lipid bilayer), step 3 may end up with some of those molecules totally or partially outside of the reference box. Furthermore, some non-assembled molecules that were initially inside the reference box (e.g., solvent molecules) may now be outside due to the translation performed in step 3. Therefore, it is necessary to move those outside molecules into the reference box by translating them an integer number of boxes in each direction, which is the final step of the algorithm. A simple way of doing this is to move each molecule so that its center is placed inside the reference box; molecules partially outside will remain partially outside, but their external portion will be minimized. Therefore, we can apply eq 5 to each atom of the system but, instead of computing the number-of-boxes displacement from its position, compute it from the position of the center of the molecule of which it is a part. Thus, for each atom *i*, its position **s**_*i*_^″^ obtained from step 3 could be translated to the new position
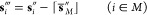
21where  is the position of the center of its containing molecule *M*, computed as in eq 10. This step would also place inside the reference box (or crossing its faces) molecules that are not part of the molecular complex (e.g., solvent molecules), for which no assembly is required.

**Figure 7:**
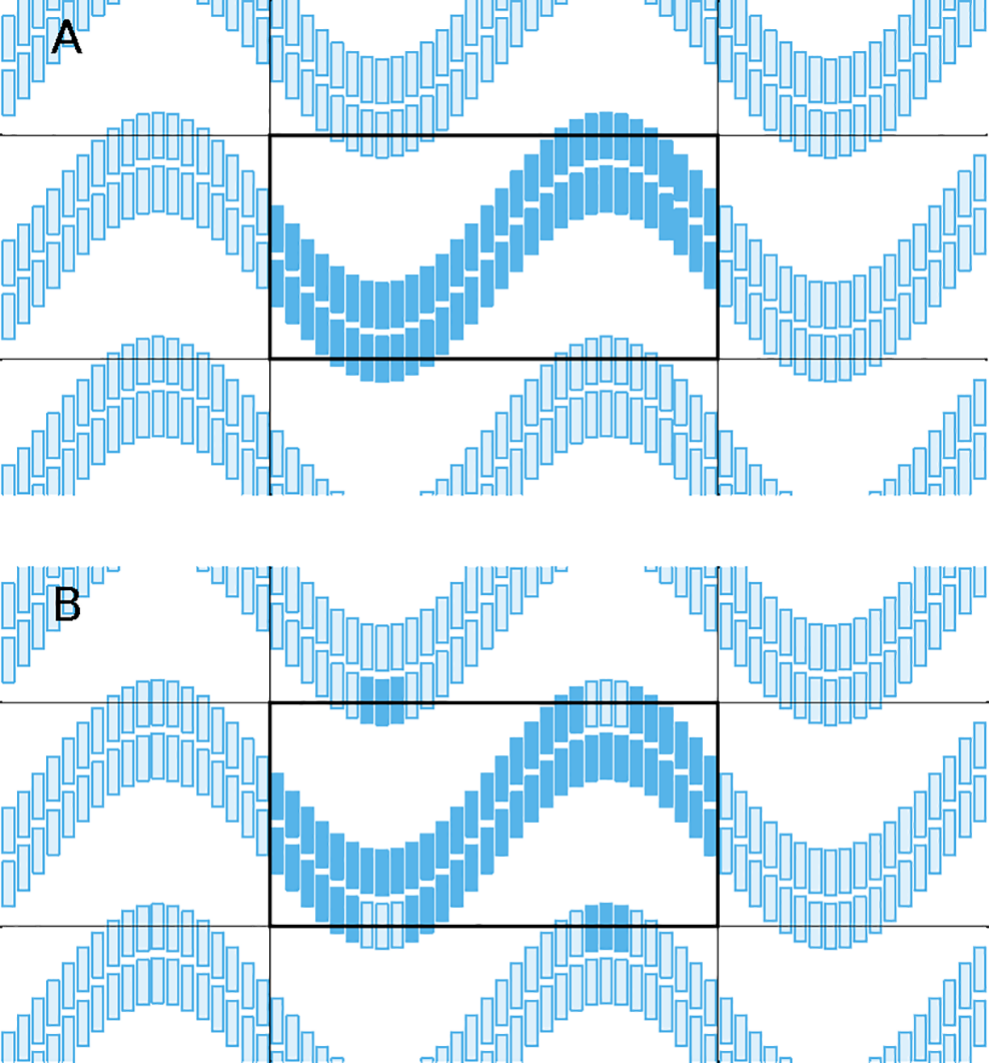
Scheme illustrating that using reference box coordinates along all directions can lead to problems. An undulating bilayer (A) can have its crests cut off by the use of reference box coordinates along the vertical direction (B).

However, in some cases, this final mapping into the reference box may not be convenient along some directions: for example, if a horizontally oriented lipid bilayer experiences undulations large enough to make its vertical range (slightly) higher than the box vertical size, we will most likely want to move the outside lipid molecules into the reference box only along the two horizontal directions but not along the vertical one because that would cut some undulation crests (Figure 7). In fact, such cases can be easily detected in step 3 because the extent of the bounding box in scaled space will be larger than 1 in one or more directions. For greater flexibility in dealing with such situations, it is thus convenient to let the user choose the sets of molecules , , and  to which each PBC mapping should be applied. Therefore, instead of applying (21) to all atoms along all directions, we apply it conditionally to the components along each direction α = *x*, *y*, *z*:
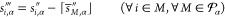
22This final step completes the algorithm, resulting in the final atomic coordinates  in physical space.

### 2.4 Workflow and Input/Output

Given the above description, the algorithm FixBox requires the following input:

- An initial set of atomic coordinates, {**r**_*i*_}, together with the primitive vectors (**b**_1_, **b**_2_, **b**_3_) of the corresponding triclinic reference box. This can be a molecular structure file in some standard format (gro, pdb, etc.). It is assumed that the provided coordinates correspond to whole molecules (i.e., not broken by the PBCs), though these are not required to be totally or even partially encompassed by the triclinic reference box.
- The specification of the molecules, that is, of which atoms *i* belong to each molecule *M*. Since only the sets of atoms per molecule are needed, the algorithm does not need a full molecular topology, which makes it more general.
- The specification of the sets of molecules to be hierarchically assembled: , etc.
- The specification of the sets of molecules to be centered along each direction: .
- The specification of the sets of molecules that should be PBC-mapped into the reference box along each direction: , , .

**Figure 8:**
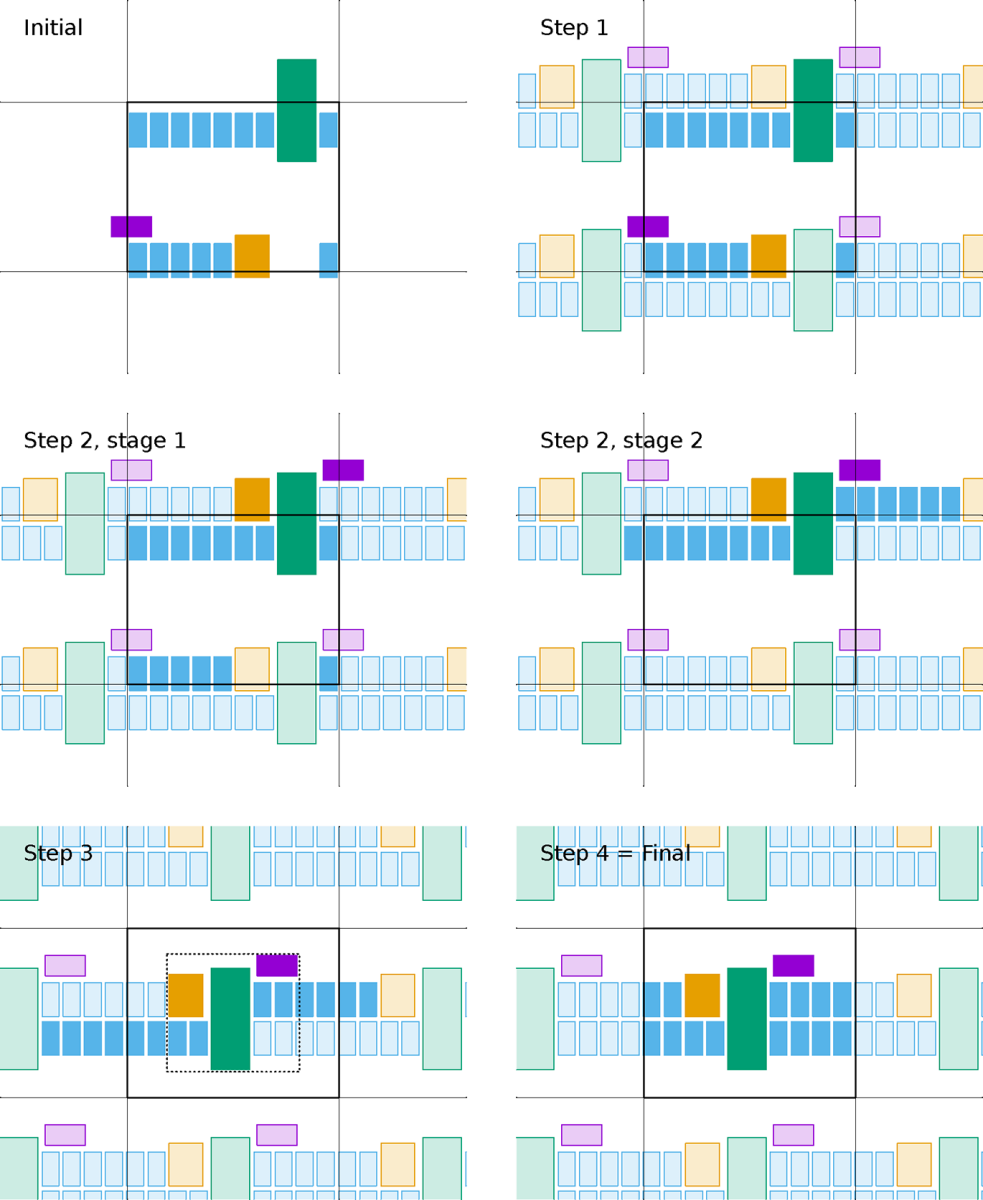
Scheme illustrating an application of the full FixBox algorithm using two assembly stages, showing the state of the system after each step. Set definitions: , , , , , . Step 1 is depicted through the introduction of the periodic images used in that step to compute the intermolecular MI-distances for all pairs of molecules. In stage 2 of step 2, it is assumed that, as in Figures 3 and 4, the nearest neighbors of the blue molecules tend to be in the same monolayer. The bounding box (dotted line) is shown in step 3.

The algorithm then performs the steps 1–4 described, resulting in the consecutive transformations {**r**_*i*_} → {**s**_*i*_} → {**s**_*i*_^′^} → {**s**_*i*_^″^} → {**s**_*i*_^‴^} → {**r**_*i*_^‴^}. The final atomic coordinates, {**r**_*i*_^‴^}, can then be saved in a new molecular structure file, keeping the original box vectors (**b**_1_, **b**_2_, **b**_3_). Figure 8 shows a complete application of the algorithm to a schematic molecular system using a two-stage assembly.

**Chart 1:**
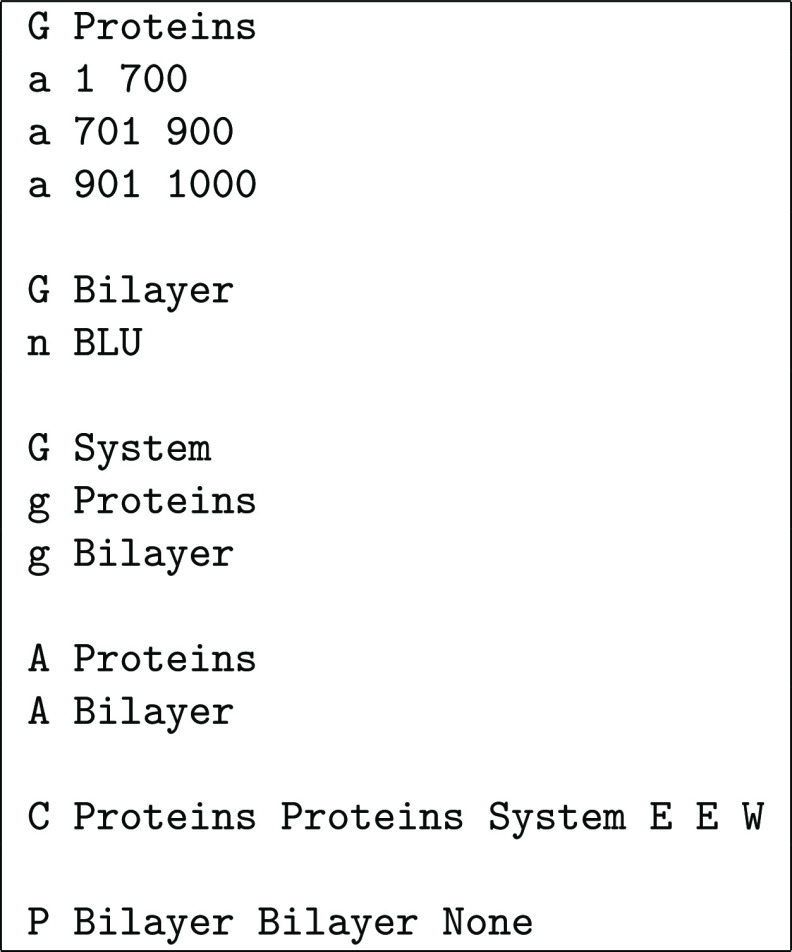
Definitions File for the Example in Figure 8

FixBox is currently implemented as a C program that reads as input a gro file^5^ with the atomic coordinates and primitive vectors plus a *definitions file* with all other required information and writes as output a gro file with the system fixed in the reference box; the input gro file may contain multiple configuration snapshots whose fixed counterparts are written to the output file. The definitions file is a very simple text file that is parsed on a single-line basis: a line starting with G is a group definition followed by single-line definitions of its elements, which may be molecules defined through atom ranges (line starting with a), single-residue molecules defined through the residue name (line starting with n), or previously defined groups (line starting with g); a line starting with A specifies the group to be used in an assembly stage, with successive such lines corresponding to , etc.; a line starting with C specifies the groups to be used for , , and , plus the corresponding error/warning flags; a line starting with P specifies the groups to be used for , , and . The syntax is illustrated in Chart 1, which shows a definitions file for the schematic example in Figure 8. Three groups are defined: Proteins contains the three proteins (green, orange, and purple, assumed to correspond, respectively, to atom ranges 1–700, 701–900, and 901–1000), Bilayer contains all the blue molecules (assumed to consist each of a single residue named BLU), and System contains the whole molecular system. As shown in Figure 8, the proteins are assembled first (with A Proteins), and then the bilayer is assembled on top of them (with A Bilayer). The proteins are then used for centering along the *x*- and *y*-axes and the whole system for centering along the *z*-axis. The final three flags on that line indicate what happens if either of the  sets extends beyond the reference box along the α-axis (α = *x*, *y*, *z*); in this case, an error (E) occurs if the proteins extend beyond the *xy*-plane and a warning (W) is issued if the system extends beyond the *z*-axis (so that undulations are allowed but noticed). Finally, bilayer molecules are moved into the reference box along the *x*- and *y*-axes but not along the *z*-axis (to avoid cutting potential undulation crests), which is indicated with the None group. There are usually alternative ways to define groups, assembly, centering, and PBCs that lead to the same final result; the user should choose the definitions that seem more intuitive or convenient in each case.

## 3 Some Applications

This section illustrates the use of the FixBox algorithm with problematic configurations of various types of systems, showing the results after each translation step of the algorithm (i.e., steps 2–4). The intermolecular distance  (see section 2.3) is used in all cases.

**Figure 9:**
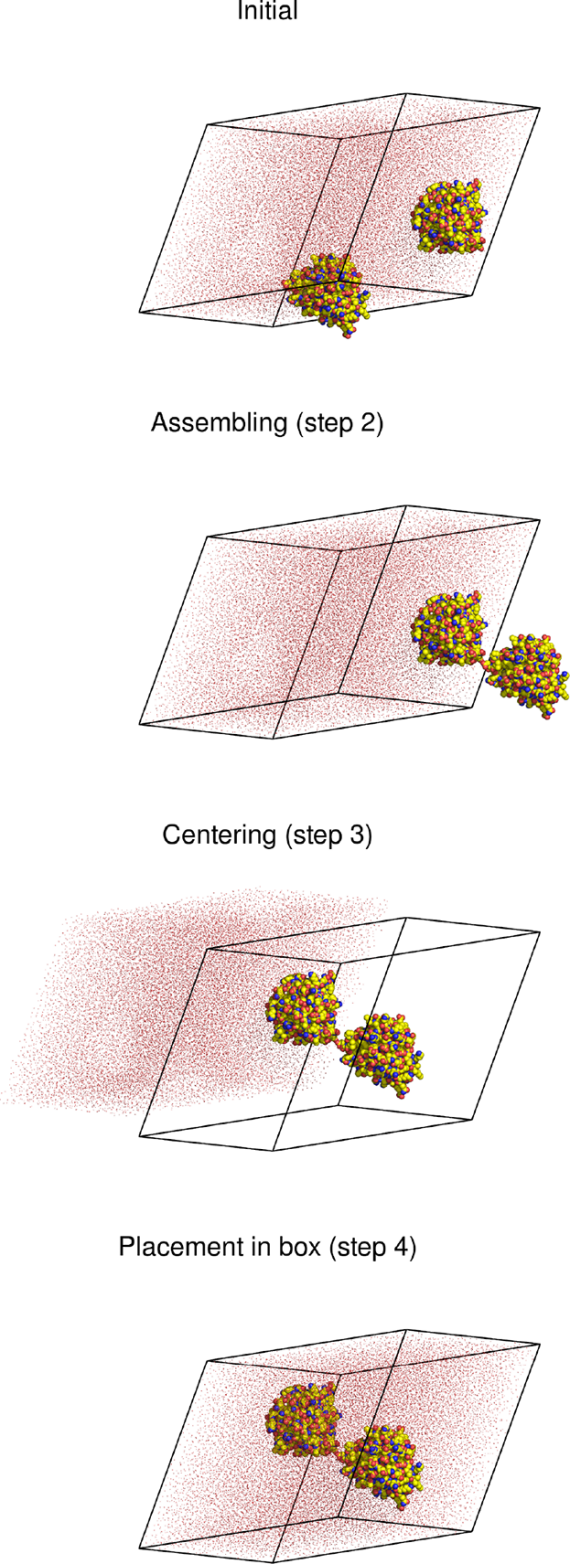
Application of the FixBox algorithm to a dimer of β-lactoglobulin (spheres) in water (dots), showing the state of the system after each translation step. The triclinic reference box is shown as black lines.

**Chart 2:**
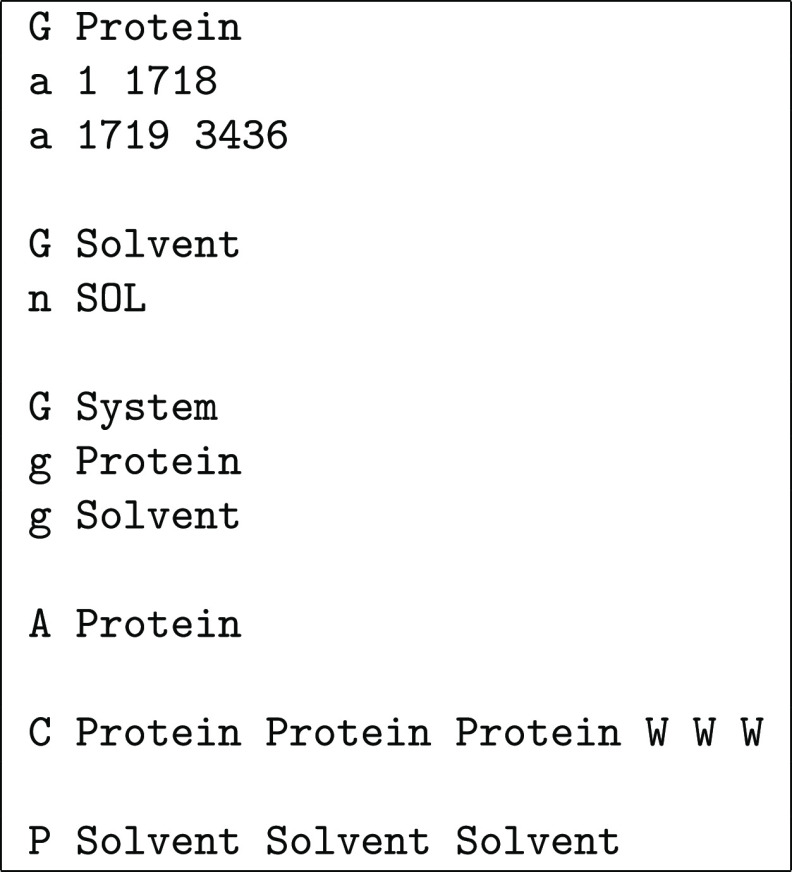
Definitions File for the Example in Figure 9

Figure 9 shows the application of the algorithm to a solvated dimer of β-lactoglobulin.^20^ This is a simple case consisting of a two-chain protein and water molecules, and a definitions file is shown in Chart 2. In order to detect potential dissociation of the dimer (e.g., when processing a trajectory), it is useful to include W flags for the centering along all directions. The triclinic box of this system is equivalent to a rhombic dodecahedron and, if necessary, the final arrangement can be transformed to that geometry using a suitable tool (e.g., GROMACS’s trjconv program^5^); but that may not be needed, as when the configuration is stripped of water and subjected to a Poisson–Boltzmann calculation.^20^

**Figure 10:**
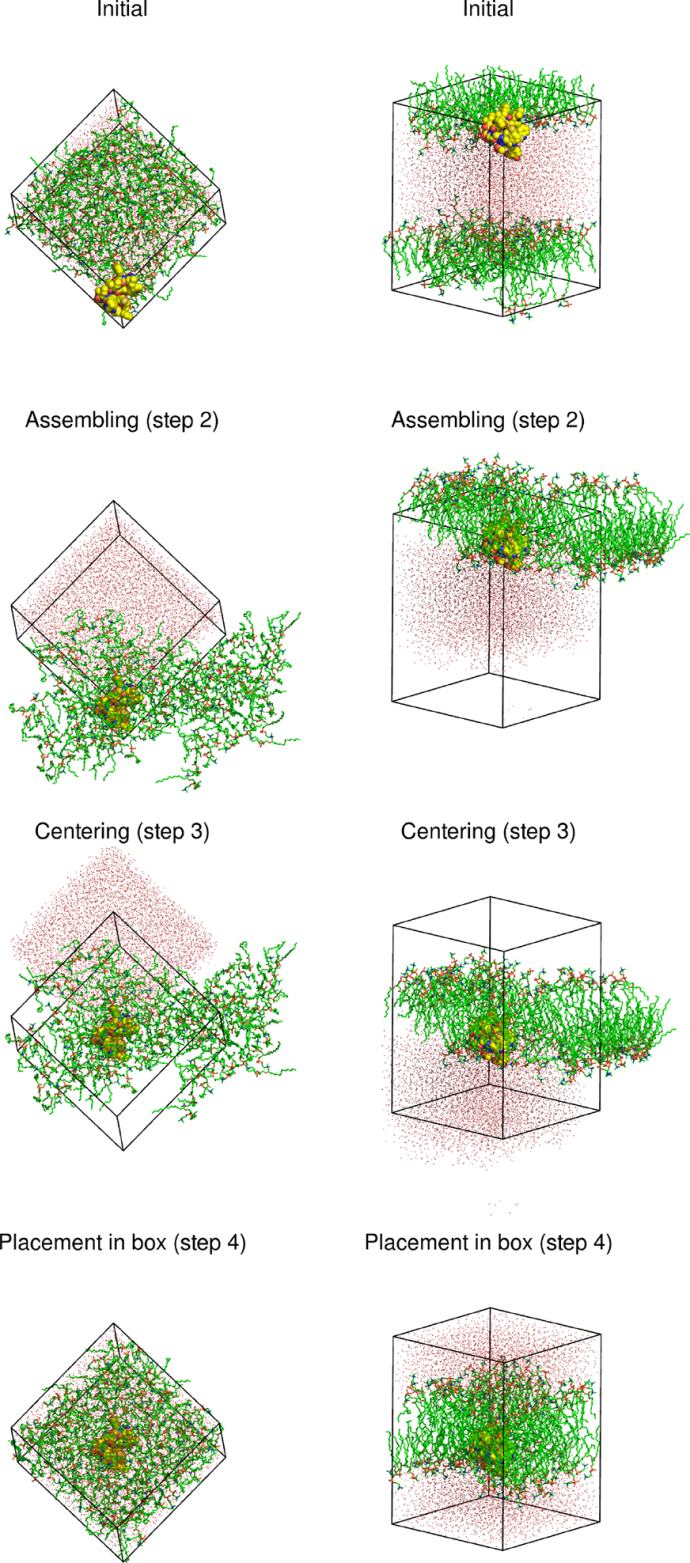
Application of the FixBox algorithm to a fusion peptide fragment of influenza hemagglutinin (spheres) interacting with a DMPC membrane bilayer (sticks) in water (dots), showing the state of the system after each translation step. The triclinic reference box is shown as black lines.

**Chart 3:**
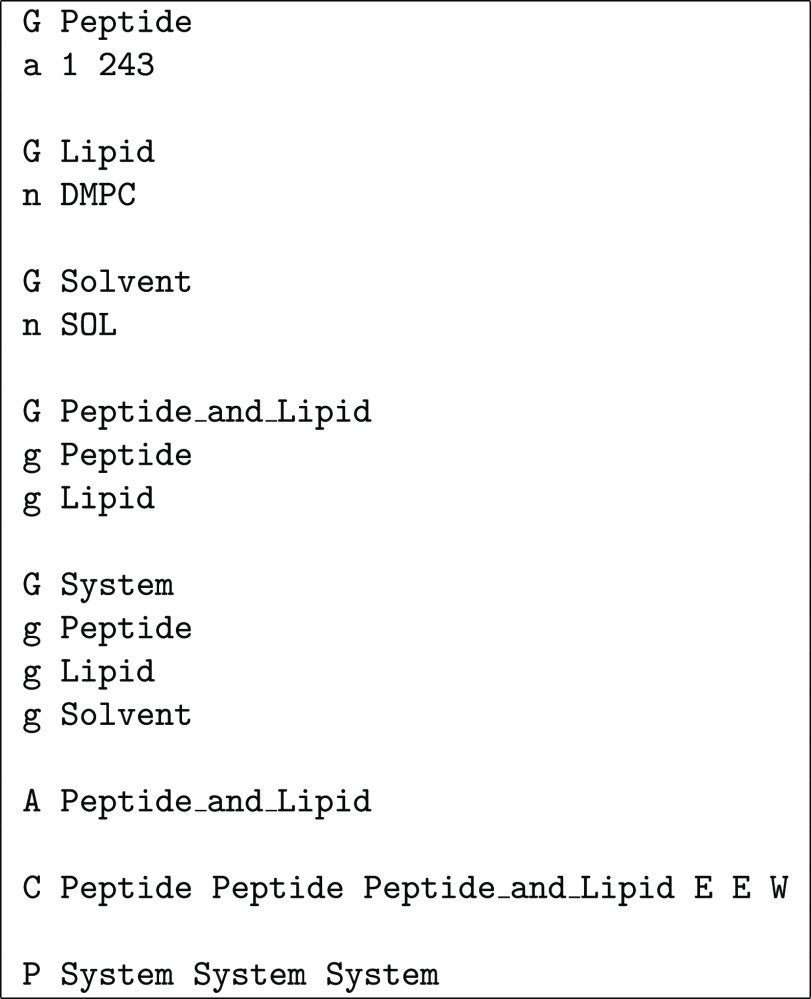
Definitions File for the Example in Figure 10

Figure 10 shows the application of FixBox to a fusion peptide fragment of influenza hemagglutinin interacting with a 1,2-dimyristoyl-*sn*-glycero-3-phosphocholine (DMPC) membrane bilayer in water.^21^ As shown in Chart 3, in this case, it is convenient to define multiple groups in order to better control what is done in each step. The assembly is applied to the peptide and the bilayer, but only the former is used for centering on the *xy*-plane. We note that the assembly step results in an almost complete separation of the two monolayers because, as already noted in section 2.3, the nearest neighbors of each lipid molecule tend to be in the same monolayer. Error flags E are used for the centering on the *xy*-plane since a failure to encompass the peptide means that it unfolded (and a wider box should have been used), while a warning flag W is used for the centering along the *z*-axis in order to detect dissociation of the peptide from the membrane.

**Figure 11:**
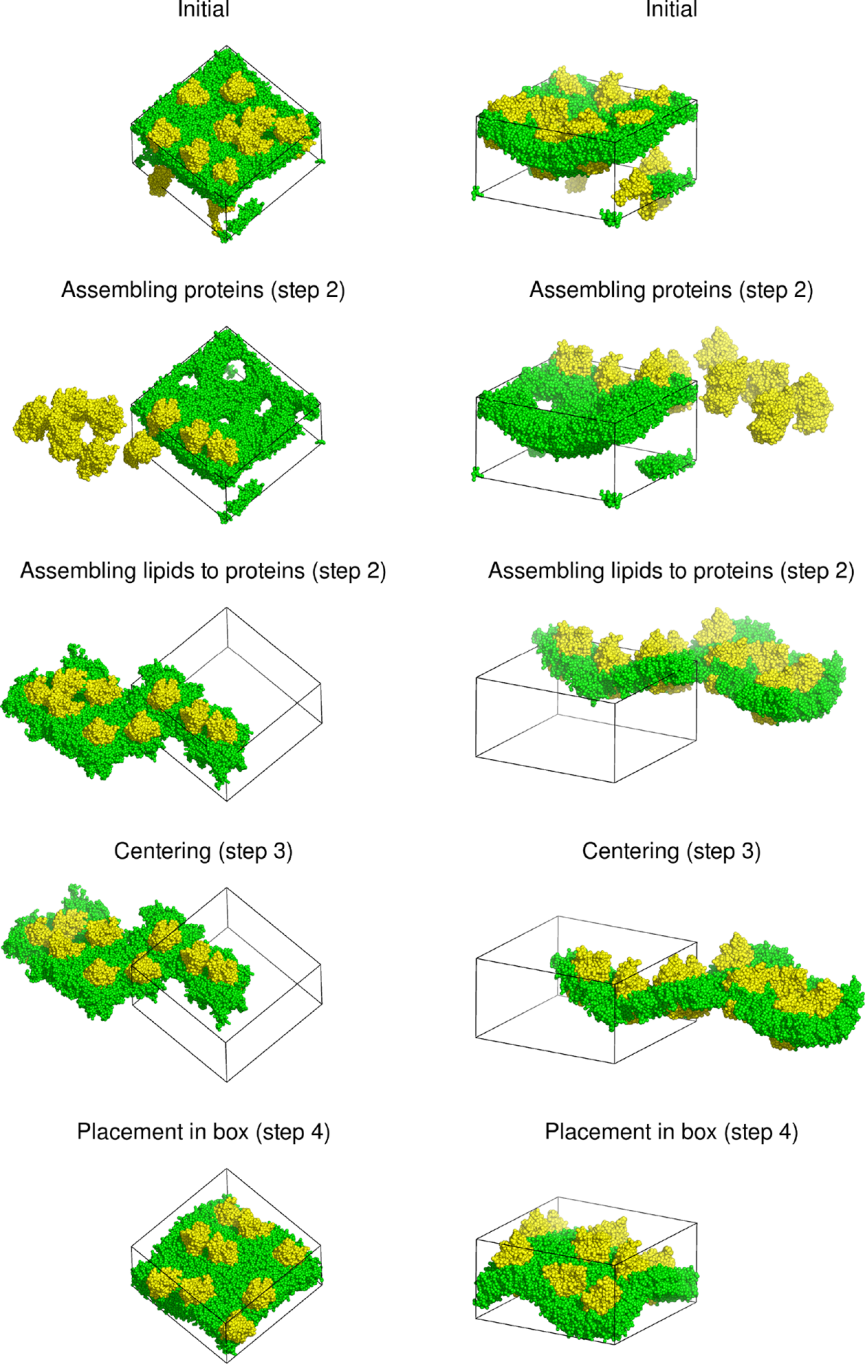
Application of the FixBox algorithm to a coarse-grained system consisting of nine cytochrome *c* oxidase molecules (yellow) on a DMPC bilayer (green), showing the state of the system after each translation step. The triclinic reference box is shown as black lines.

**Chart 4:**
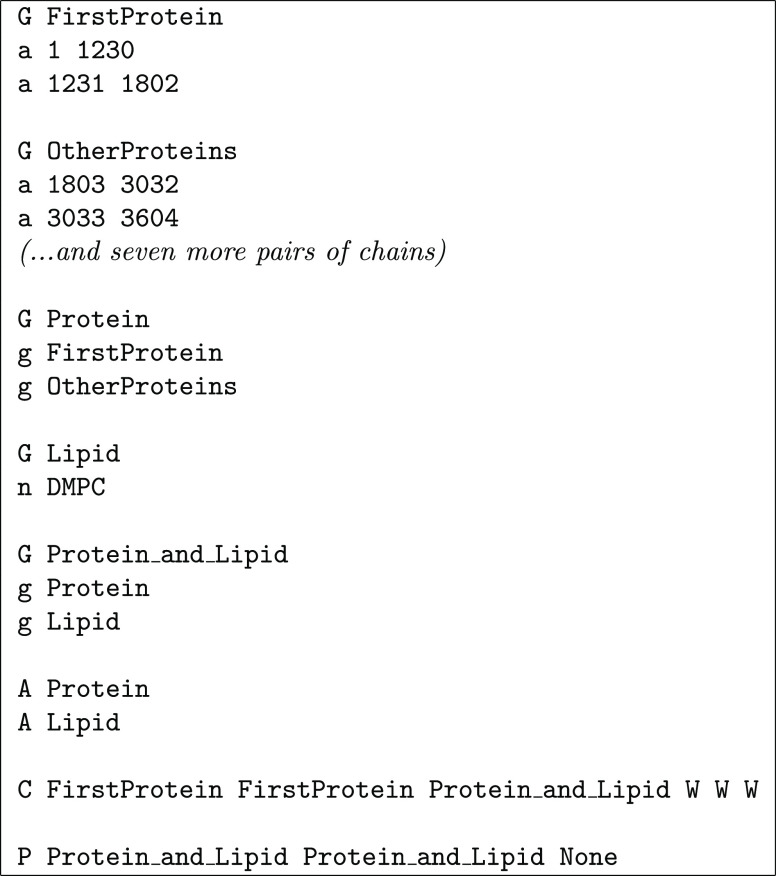
Definitions File for the Example in Figure 11

A more challenging membrane system is shown in Figure 11, containing a coarse-grained model of nine cytochrome *c* oxidase molecules (subunits I and II) embedded on a DMPC bilayer that displays substantial undulations. As seen in Chart 4, multiple groups are defined in order to better control what is done in each step. The assembly is done in two stages to avoid the problem discussed in section 2.3. Centering on the *xy*-plane is done with a group consisting of a single cytochrome *c* oxidase (FirstProtein), while the set of all proteins and lipids is used to center along the *z*-axis; in this case, warning flags (W) were used along all directions (the first two would signal dissociation of FirstProtein and the third would signal undulations). No molecules are moved into the reference box along the *z*-axis to avoid cutting the undulation crests, which are indeed present in the configuration being shown. The strategy adopted for centering on the *xy*-plane is arguable, opting for splitting again a “molecular complex” that, instead of forming a single aggregate, displays several small ones. The alternative to center the whole Protein group on the *xy*-plane would result in a bounding box much wider than the reference box, which, unlike the situation along the *z*-axis, would not be a satisfactory solution. This illustrates that FixBox is at present sub-optimal when dealing with multiple aggregates.

**Figure 12:**
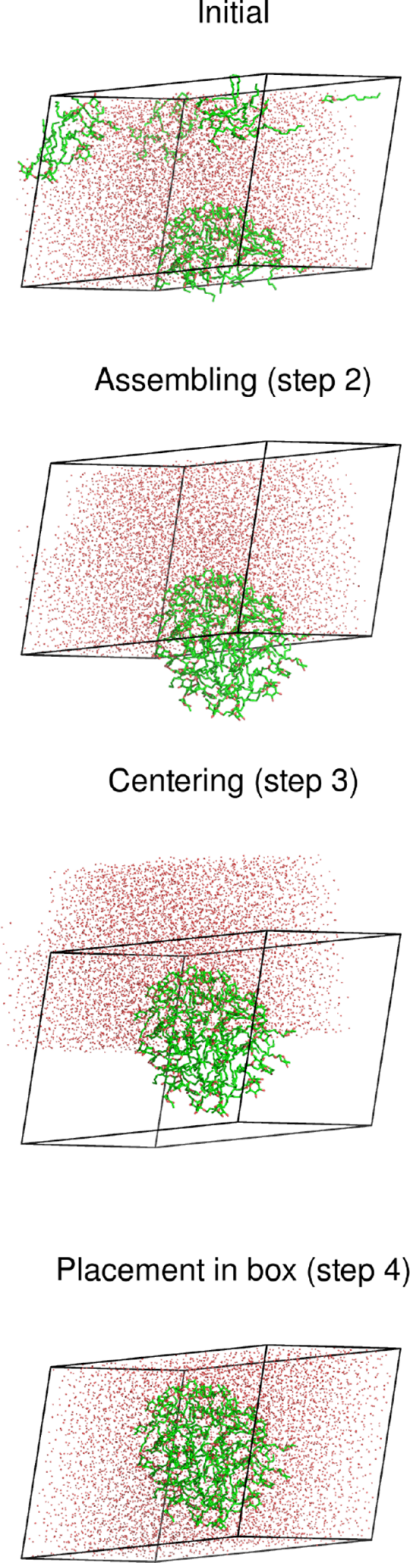
Application of the FixBox algorithm to a glycoside micelle (sticks) in water (dots), showing the state of the system after each translation step. The triclinic reference box is shown as black lines.

**Chart 5:**
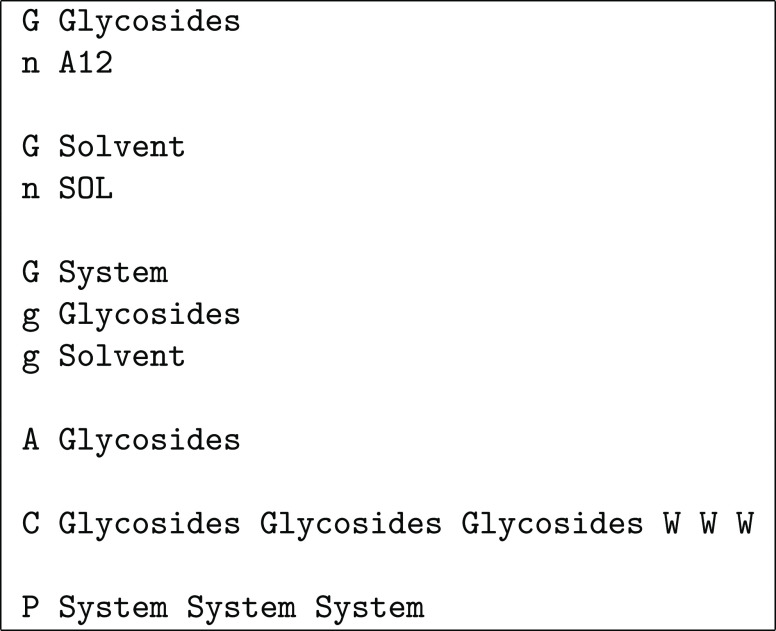
Definitions File for the Example in Figure 12

Figure 12 shows a micelle composed of amphiphilic glycoside molecules (compound 1 in ref (22)) solvated in water. This is a simple example in which the complex to be assembled is naturally an aggregate, requiring a simple definitions file (Chart 5). The warning flags make it easy to detect if the glycoside molecules are in fact aggregated.

The fact that FixBox does not make direct use of a topology can also be occasionally exploited. For example, if a molecular complex contains a strictly periodic (“infinite”) molecule, the algorithm can be easily misled to do what we want by treating its structural units as distinct molecules, which, in analogy with a lipid bilayer, would then be assembled in a proper configuration that preserves the strict molecular periodicity.

## 4 Concluding Remarks

The FixBox algorithm presented here provides a general and easy-to-use method to fix a molecular system that has been “broken” during a simulation with PBCs. Its new features include multi-stage reassembly of a molecular complex based on the shortest distance between its molecules, centering along different directions using different sets of molecules, centering using bounding boxes, and PBC-wrapping along different directions using different sets of molecules; together, these allow for a level of “box fixing” not usually attainable with available tools. FixBox can be used to process simulation trajectories prior to analysis or as part of a more complex automated procedure in which box fixing is required (e.g., web servers for Poisson–Boltzmann calculations^23,24^ or some constant-pH MD methods^3,4^). The use of a unified triclinic framework makes the algorithm agnostic with respect to box geometry (only the box primitive vectors must be provided in addition to the atomic coordinates), and the fixing of the system is based on a simple set of definitions of its assembling parts and their intended arrangement (provided in a definitions file). FixBox is intended for general use as illustrated in section 3, but it is not expected to deal optimally with multiple molecular aggregates (e.g., a system with several micelles), although it might make a reasonable job in some such cases. A proper understanding of the algorithm and a careful choice of definitions file should make it possible to deal with a wide range of systems. The present article provides also a formal framework that can be useful for the discussion and presentation of this type of algorithms.

## 5 Data and Software Availability

The current open source implementation of FixBox is available at https://www.itqb.unl.pt/simulation/in-house-software, both standalone and as part of our in-house implementation of the stochastic titration method for constant-pH MD,^3,4^ which was the main drive for its development.
